# Longitudinal changes in bone and mineral metabolism after cessation of cinacalcet in dialysis patients with secondary hyperparathyroidism

**DOI:** 10.1186/s12882-018-0910-9

**Published:** 2018-05-15

**Authors:** Irene Ruderman, Edward R. Smith, Nigel D. Toussaint, Tim D. Hewitson, Stephen G. Holt

**Affiliations:** 1 Department of Nephrology, The Royal Melbourne Hospital, 300 Grattan St, Parkville, Victoria 3050 Australia; 2 Department of Medicine (RMH), The University of Melbourne, Melbourne, Victoria Australia

**Keywords:** End-stage kidney disease, Secondary hyperparathyroidism, Dialysis, Calciprotein particles, Parathyroid hormone, Cinacalcet

## Abstract

**Background:**

The calcimimetic agent cinacalcet is effective for the management of secondary hyperparathyroidism (SHPT) in dialysis patients. Changes to reimbursement of cinacalcet in Australia provided an opportunity to assess effects of medication cessation on biochemical and clinical outcomes in dialysis patients, including changes to novel biomarkers such as calciprotein particles (CPP). CPP are nanoparticles of mineral and protein in the circulation associated with increased vascular calcification in patients with chronic kidney disease.

**Methods:**

Dialysis patients from a single center who ceased cinacalcet between August 2015 and March 2016 were included in a prospective observational study. Bloods were taken at the time of cessation of cinacalcet and at 1, 6 and 12 months. Clinical and biochemical outcomes were compared with an age- and gender-matched cohort of cinacalcet-naïve dialysis patients.

**Results:**

Sixty-two patients participated in the study. Mean age was 69.6 ± 13.2 years. Biochemical changes over 12 months following cessation of cinacalcet included an increase in serum parathyroid hormone (PTH) (42.2 [IQR 27.8–94.6] pmol/L to 114.8 [83.9–159.1] pmol/L [*p* < 0.001]), serum calcium (2.31 ± 0.21 mmol/L to 2.46 ± 0.14 mmol/L [*p* < 0.001]) and primary CPP (CPP-I) (*p* = 0.002). Changes in CPP were associated with an increase in PTH (*p* = 0.007), calcium (*p* = 0.002) and ferritin (*p* = 0.02) but a reduction in serum albumin (*p* = 0.001). Over the 12-month period, there were two fractures, five cardiovascular events, one episode of calciphylaxis, and one parathyroidectomy, with a mortality rate of 19% (*n* = 13).

**Conclusion:**

Uniquely we report the effects of cinacalcet withdrawal in a real world setting with demonstrated increases in PTH, serum calcium and CPP subsets, novel CKD-MBD related factors, over a 12-month period.

## Background

Abnormalities in bone and mineral metabolism, encompassed by the term ‘chronic kidney disease - mineral and bone disorder’ (CKD-MBD) [1], play a significant role in vascular calcification and increased cardiovascular risk in patients with chronic kidney disease (CKD) [2]. Progressive changes in mineral homeostasis with disruption of normal calcium and phosphate balance are associated with changes in the phosphaturic hormones like fibroblast growth factor 23 (FGF23) and parathyroid hormone (PTH), and abnormalities in vitamin D metabolism, and these result in complications of secondary hyperparathyroidism (SHPT) [3]. SHPT has been linked with pathology [4] including; bone disease [5], hip fractures [6], myocardial hypertrophy and dysfunction [7], disturbances in lipid [8] and glucose [9] metabolism, anemia [4] and vascular calcification [10].

Vascular calcification in patients with CKD results from several interlinked mechanisms that involve aberrant bone metabolism, inflammatory pathways and dysregulation of endogenous calcification inhibitors [11]. One potent inhibitor of endogenous calcification is fetuin A, a highly conserved and ubiquitous phosphoprotein present in all mammals which forms nanoparticles with calcium and phosphate in the circulation forming calciprotein particles (CPP). CPP may provide an important pathway for transporting mineral nanocrystals around the body and clearance in the circulation is by macrophages [12]. Initially these nano-sized particles are present in amorphous calcium-phosphate form (primary CPP or CPP-I), however chronic dysregulation of mineral metabolism may result in accumulation and ripening of these particles into larger crystalline calcium phosphate (secondary CPP or CPP-II), possibly resulting in toxicity [13–15]. Levels of CPP are significantly higher in patient cohorts known to develop premature ageing and arterial calcification, such as patients with CKD (especially those on dialysis) [14, 16] and those with inflammatory diseases [16], compared to healthy controls.

Higher levels of CPP are a predictor of all-cause mortality in patients with CKD [17], and have been associated with increased vascular calcification [18] and calciphylaxis in this population [16, 19]. In patients on dialysis, reduction of PTH by parathyroidectomy or calcimimetics also results in a reduction in CPP [20], suggesting that abnormal bone metabolism is associated with mineral stress, leading to an imbalance in production and removal of CPP. Detection of elevated levels of CPP in the circulation in pathology may therefore serve as a novel biomarker of mineral stress and cardiovascular risk.

The calcimimetic agent cinacalcet is commonly used in the management of SHPT, with multiple studies reporting clinical efficacy of this therapy to reduce PTH levels [21, 22]. This medication was approved for treatment of SHPT in Australia in 2007, with associated government reimbursement, and has been widely prescribed to control biochemical changes associated with moderate to severe SHPT in patients on dialysis. Following the publication of the Evaluation of Cinacalcet Hydrochloride Therapy to Lower Cardiovascular Events (EVOLVE) study [23] which failed to show an effect for cinacalcet vs. placebo in unadjusted intention-to-treat analysis of the primary composite end-point (time to death, myocardial infarction, hospitalization for unstable angina, heart failure, or a peripheral vascular event), the pharmaceutical benefits advisory committee in Australia withdrew reimbursement for this medication. Cinacalcet is now available only on private prescription and at a significant cost to the patient. The effect of this change meant that cinacalcet was withdrawn in the majority of patients previously prescribed, and this provided a unique opportunity to study changes in markers of bone and mineral metabolism, including CPP, in patients with SHPT.

## Methods

### Study design

This was a single-center prospective observational study performed at The Royal Melbourne Hospital. The aim was to assess the impact of withdrawal of cinacalcet in patients on dialysis over a 12-month period. The study was approved by the Melbourne Health Human Research Ethics Committee (#HREC 2015.180) and patient enrollment commenced in August 2015 with completion in March 2016.

### Study cohort

Patients receiving dialysis at The Royal Melbourne Hospital or affiliated satellite dialysis units were eligible for recruitment, and written informed consent was obtained from all participants at study commencement. Inclusion criteria included patients with the ability to provide informed consent, aged over 18 years old and prescribed cinacalcet therapy for clinical features of severe SHPT, not adequately controlled with active vitamin D therapy, and a PTH level greater than nine times the upper limit of normal as per the Kidney Disease: Improving Global Outcome (KDIGO) CKD-MBD guidelines [1]. There were no specific exclusion criteria. Starting doses of cinacalcet were 30 mg/day and doses had been titrated to achieve symptom control and a PTH target within the KDIGO guideline suggested range. A detailed medication history was recorded for patients at each visit, and date of cinacalcet cessation was documented in the patient’s medical records. All patients provided written informed consent before enrollment and the study was conducted in accordance with the Declaration of Helsinki.

No specific protocol existed for the management of SHPT following cinacalcet withdrawal. Treatment was based on usual clinical care for SHPT including reduction of serum phosphate and administration of active vitamin D therapy with avoidance of hypocalcemia. Patients were referred for surgical parathyroidectomy if they had a persistently elevated PTH above the KDIGO guidelines, evidence of high turnover bone disease or symptoms of severe PTH. Dialysate calcium in hemodialysis and peritoneal dialysis patients remained unchanged following cinacalcet withdrawal.

### Control cohorts

Two control cohorts were used in this study to the potential effects of temporal changes unrelated to drug cessation. The first was a historical age-, gender- and dialysis vintage- matched control cohort who were cinacalcet naïve. We compared demographic features and biochemical parameters over a 12-month period between the control cohort and the cinacalcet withdrawal cohort from July 2015 to July 2016. This control cohort was identified from the Nephrology database at The Royal Melbourne Hospital, and all patients included had no history of prior cinacalcet use. The second control group included a subset of thirteen cinacalcet naïve dialysis patients from the historical control cohort that were age and dialysis modality matched, who had serum samples collected at baseline (June 2017) and after 6 months for CPP analysis. The purpose of this control group was to assess whether a rise in CPP observed in the intervention group reflected a response to drug cessation or a more generic rise in CPP that would be observed in any group of chronic dialysis patients with progressive disease over time. Management of CKD-MBD in the control cohort was as per usual standard care for SHPT, involving treatment of hyperphosphatemia and use of active vitamin D therapy as indicated by the treating clinician.

### Study end points

The primary endpoint was change in CPP following cessation of cinacalcet therapy over a 12-month period. Secondary biochemical outcome measures included serum changes in PTH, calcium, phosphate, alkaline phosphatase (ALP), ferritin and C-reactive protein (CRP), as well as hemoglobin.

### Biomarker assessment

Serum was collected in all patients at baseline, whilst still being administered cinacalcet and then following cessation of cinacalcet at 1 month, 6 months and 12 months. Samples were collected to measure the following parameters: total CPP, CPP-I, CPP-II, albumin, calcium, phosphate, PTH, hemoglobin, CRP, ferritin, and ALP. Serum calcium level was adjusted as follows if serum albumin was < 40 g/L: corrected serum calcium (mmol/L) = measured serum calcium (mmol/L) + 0.02 x (40 - serum albumin (g/L)). For CPP analysis blood was collected into 6 ml plain tubes using standard phlebotomy techniques. Blood samples were allowed to stand for 60 min and then centrifuged at 3000 g for 15 min at room temperature. Aliquots were stored at − 80 C until batched analysis.

### CPP evaluation using flow cytometry

**Fig. 1:**
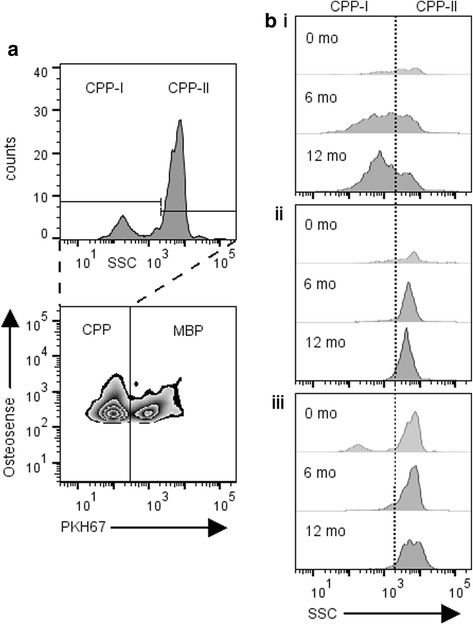
Schematic of flow cytometry gating strategy. **a** Represents cytograms showing dual Osteosense and PKH67 staining on mineral-containing nanoparticles (**b**) Histograms show gated populations and their respective volumetric measurements (counts/μL). CPP-I, primary calciprotein particle; CPP-II, secondary calciprotein particle

A novel method to evaluate CPP using flow cytometry has recently been published by our group [15]. Briefly, batched patient samples were run on a BD FACSVerse flow cytometer using high sensitivity fluidics. The instrument was operated using BD FACSSuite software (version 1.0.5). Raw FCS files were acquired and imported into FlowJo LLC version 10.1 revision 3 (Ashland, Oregon, USA) for analysis. OsteoSense 680EX fluorescence was detected with a red 640 nm laser. Acquisition settings were held constant for all samples (60 s or 30,000 events). All measurements were displayed in logarithmic scale and signal stability was assessed in real-time using SSC-H vs. time plots. The gating strategy for CPP was set empirically, as described in Fig. 1.

### Statistical analysis

Baseline characteristics are reported as mean+/− standard deviation (SD) or, when appropriate, median (interquartile range [IQR]) for continuous variables and as a number and percentage for categorical variables. Paired t test and Wilcoxon signed-rank test were used for between group comparisons. Categorical variables were analyzed with the chi-square test. Continuous variables were compared with independent samples paired t test if normally distributed or with Mann-Whitney U test if the distribution was skewed. Mixed linear effect modelling was used to determine differences within groups over the study period, with the variables selected a priori*,* based on known regulatory relationships. CPP and PTH were natural log-transformed (Ln) for the purposes of these analyses. All mixed-effect model analyses were performed allowing for the random effect of different pathology collection times and locations. Two-tailed *P* values < 0.05 were considered statistically significant. Analyses were performed using SPSS software for Macintosh version 21 (IBM SPSS, Chicago, IL).

## Results

### Demographics and clinical characteristics

**Fig. 2:**
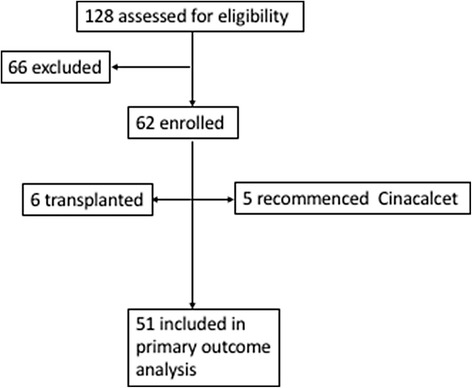
Participant flow diagram

One hundred and twenty-eight patients on dialysis were being administered cinacalcet during the recruitment period of August 2015 to March 2016 and were approached to participate in the study. Figure 2 describes the participant flow. Sixty-six were excluded either due to patient or physician preference as many of these patients had a supply of medication remaining and continued on therapy. Sixty-two patients were enrolled in the study, and their cinacalcet cessation date was documented in medical records. Six patients received a kidney transplant and five re-commenced cinacalcet therapy via an industry sponsored special-access scheme during the 12-month follow up period. Fifty-one patients were included in the primary outcome analysis.

Baseline characteristics of study participants and the contemporaneous age- and gender-matched cinacalcet-naïve dialysis controls are shown in Table 1. Only dialysis modality was significantly different between the two groups, with more patients in the cinacalcet withdrawal cohort on hemodialysis, 86% vs 57%, (*p* = 0.001). The mean age and time on dialysis were 69.9±13.2 years and 7.1±3.6 years respectively in the cinacalcet withdrawal cohort. Over 50% of participants were male, 45% had a history of diabetes and 76% had hypertension. The main etiology of CKD was diabetic nephropathy.

**Table 1:** Patient demographics and clinical characteristics

Demographic	Cinacalcet withdrawal patients (*n* = 51)	Control patients (*n* = 51)	*p* value
Age, years	69.6 (13.2)	68.6 (12.9)	ns
Gender (male)	28 (55)	28 (55)	ns
Dialysis modality (HD)	44 (86)	29 (57)	*p* = 0.001
Time on dialysis, years	7.1 (3.6)	6.7 (4.1)	ns
Diabetes	23 (45)	23(45)	ns
Hypertension	39 (76)	33 (65)	ns
Ischemic heart disease	23 (45)	18 (35)	ns
Peripheral vascular disease	8 (15)	15 (29)	ns
Current or ex-smoker	16 (31)	17 (33)	ns
Previous transplants	4 (8)	2 (4)	ns
Parathyroidectomy	1 (2)	5 (10)	ns
Previous fractures	7 (13)	4 (8)	ns
Aetiology of renal disease			
Diabetic nephropathy	14 (27)	18 (35)	ns
GN	9 (17)	12 (23)	ns
PKD	3 (6)	4 (8)	ns
Other	25	17	ns
Events during follow up			
Cardiac arrest	5	3	ns
Parathyroidectomy and referral for surgery	5	1	ns
Fracture	2	5	ns
Calciphylaxis	1	0	ns
Deaths	13	10	ns
Hypercalcemia at baseline	3	1	ns
Hypercalcemia at 6 months	9	0	*p* = 0.007
Hypercalcemia at 12 months	3	0	ns

Data presented as number (percent), or mean (standard deviation)

Hypercalcemia if Ca > 2.6 mmol/L

Abbreviations: *HD* hemodialysis, *GN* glomerulonephritis, *PKD* polycystic kidney disease

In the 12 months following withdrawal of cinacalcet therapy there were two lower limb fractures, one parathyroidectomy with three patients referred for parathyroid surgery, and one episode of calciphylaxis. Thirteen patients died during the study period, with deaths due to cardiovascular causes (*n* = 5), withdrawal from dialysis (*n* = 3) and other causes including malignancy and infection (*n* = 4). In the control group there were five fractures, one parathyroidectomy and no episodes of calciphylaxis. Of the 10 deaths in the control group, three were due to cardiovascular causes.

A change in prescribing patterns following cinacalcet withdrawal were observed. At baseline 76% of patients (*n* = 39) were on calcitriol, which reduced to 57% (*n* = 29) by 12 months (*p* = 0.03). Four patients were commenced on calcitriol de novo. Calcitriol doses increased over the study period (1.3 mcg/week versus 1.7 mcg/week at baseline and 12 months respectively, *p* = 0.04). Of 21 patients on a calcium-based phosphate binder at baseline, 12 continued this therapy at 12 months (*p* = 0.05). Seventy-six percent of patients (*n* = 39) were on a non-calcium based phosphate binder at baseline, 59% of patients (*n* = 30) remained on therapy at study completion (*p* = 0.06). No patients were on magnesium supplementation. There were 12 episodes of hypercalcemia (serum corrected calcium > 2.60 mmol/L), with 9 episodes occurring by 6 months. There were no episodes of hypercalcemia in the control arm. The dosage or number of patients taking nutritional vitamin D supplementation did not significantly change over the year (12 at baseline versus 10 at 12 months, *p* = 0.63).

### Biochemical outcomes

Baseline levels of PTH, phosphate, calcium and ALP were similar across the two groups (Table 2). In the cinacalcet withdrawal cohort, there was an increase in PTH from 42.2 pmol/L (27.8–94.6 pmol/L) at baseline to 114.8 pmol/L (83.9–159.1 pmol/L) at 12 months (*p* < 0.001). The highest rate of change in PTH occurred at 1 month with a mean PTH increase of 93% from baseline (*p* < 0.001). Serum calcium also increased from 2.31±0.21 mmol/L to 2.46±0.14 mmol/L over 12 months (*p* < 0.001). There was no change in PTH or serum calcium in the control group over 12 months. Phosphate remained unchanged in the cinacalcet withdrawal group (*p* = 0.8) and the control group (*p* = 0.6) over the 12-month study period. Table 2 summarizes baseline, 6- and 12-month values for biochemical outcomes in the control and cinacalcet withdrawal cohorts.

**Table 2:** Biochemical changes over 12-month period

Biochemistry	Cinacalcet withdrawal patients (*n* = 51)	Control patients (*n* = 51)	*p* value
PTH baseline, (pmol/L)	42.2 [27.8–94.6]	44 [28.5–60.9]	*p* = 0.98
PTH 6 months, (pmol/L)	103.9 [63.4–122.2]	41 [28.2–67.8]	*p* < 0.005
PTH 12 months (pmol/L)	114.8 [83.9–159.1]	41.3 [28.5–69.7]	*p* < 0.005
Calcium baseline (mmol/L)	2.31 (0.21)	2.32 (0.15)	*p* = 0.8
Calcium 6 months (mmol/L)	2.48 (0.21)	2.28 (0.18)	*p* < 0.005
Calcium 12 months (mmol/L)	2.46 (0.14)	2.32 (0.14)	*p* < 0.005
Phosphate baseline (mmol/L)	1.72 (0.55)	1.56 (0.42)	*p* = 0.14
Phosphate 6 months (mmol/L)	1.75 (0.47)	1.64 (0.56)	*p* = 0.3
Phosphate 12 months (mmol/L)	1.77 (0.58)	1.6 (0.49)	*p* = 0.14
ALP baseline (IU/L)	141.4 (61)	129.8 (91.8)	*p* = 0.45
ALP 6 months (IU/L)	155.5 (61)	126 (75.5)	*p* = 0.04
ALP 12 months (IU/L)	161.7 (72.9)	121.5 (45)	*p* = 0.003
Albumin baseline (g/L)	35 (4.2)	35.7 (4.3)	*p* = 0.84
Albumin 6 months (g/L)	34 (4.2)	34.9 (4.6)	*p* = 0.97
Albumin 12 months (g/L)	33 (4.5)	34.3 (5)	*p* = 0.94
CRP baseline (mg/L)	6.3 [4.6–19.6]	5 [2–16]	*p* = 0.312
CRP 6 months (mg/L)	7 [3.3–12.3]	4.2 [2–18]	*p* = 0.37
CRP 12 months (mg/L)	7.7 [3–29.5]	6.3 [2–19]	*p* = 0.41
Ferritin baseline (ug/L)	235.5 [102.3–322.3]	229 [140.8–399.3]	*p* = 0.23
Ferritin 6 months (ug/L)	209.5 [80–331.3]	232.5 [114–316]	*p* = 0.86
Ferritin 12 months (ug/L)	274.5 [120–383.5]	221.5 [138.5–322.8]	*p* = 0.64
Hemoglobin baseline (g/L)	112.7 (10.4)	110 (14)	*p* = 0.13
Hemoglobin 6 months (g/L)	111.6 (10.7)	107 (18)	*p* = 0.37
Hemoglobin 12 months (g/L)	109 (14)	114 (14)	*p* = 0.09
Bicarbonate baseline (mmol/L)	23 (3)	24 (3.7)	*p* = 0.53
Bicarbonate 6 months (mmol/L)	23.1 (3)	23.9 (3)	*p* = 0.9
Bicarbonate 12 months (mmol/L)	23.6 (3.2)	24 (3)	*p* = 0.81
25[OH]D baseline (nmol/L/L)	43[33–68]	52[42–60]	*p* = 0.52
25[OH]D 6 months (nmol//L)	49[32–68]	66[51–89]	*p* = 0.1
25[OH]D 12 months (nmol/L)	53[28–70]	60[42–97]	*p* = 0.36

Data presented as number (percent), mean (standard deviation) or median [interquartile range]

Abbreviations: *ALP* alkaline phosphatase, *CRP* C-reactive protein, *PTH* parathyroid hormone, *25[OH]D* 25-hydroxy vitamin D

**Fig. 3:**
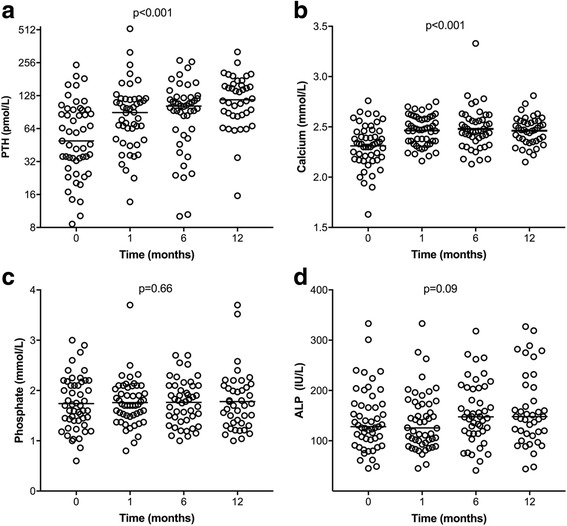
Changes in biochemical mineral markers over 12 months following cinacalcet withdrawal. Changes in (**a**) parathyroid hormone, (**b**) calcium, (**c**) phosphate and (**d**) alkaline phosphatase over a 12-month period summarized in box plots (median, lower and upper quartile, and outliers). Changes between time points measured using mixed linear effect model. *P* value denotes trend over 12-month period

A trend towards increased ALP (141.4±61 IU/L at baseline to 161.7±72.9 IU/L at 12 months) was seen in the cinacalcet withdrawal cohort over the study period but did not reach statistical significance (*p* = 0.09). Inflammatory markers including CRP and the positive acute-phase reactant ferritin remained unchanged over 12 months, however there was a modest decrease in serum albumin from 35±4.2 g/L to 33±4.5 g/L, *p* = 0.03. This trend was not observed in the control group. Hemoglobin and serum bicarbonate were also equivalent in both groups over the study period. Changes in biochemical markers in the cinacalcet withdrawal group are presented in Fig. 3.

### CPP assessments

CPP assessment and characterization was performed using a novel flow cytometry method published by our group [15]. Four out of 51 study patients were excluded from final CPP analysis due to sample instability and unknown analytical interference. Table 3 describes the changes in CPP in the cinacalcet withdrawal group, including patients who received a transplant or re-started therapy during the study period. Total CPP at baseline were 3.3 × 10^4^/μL (IQR: 1.5 × 10^4^/μL to 5.7 × 10^4^/μL), predominantly as CPP-II at baseline (61% of total CPP) and 12 months (58% of total CPP), *p* = 0.06.

**Table 3:** CPP levels in study cohort and control dialysis group

CPP/μL	Cinacalcet withdrawal patients (*n* = 47)	Transplanted patients (*n* = 6)	Patients re-started on cinacalcet (*n* = 5)	*P* values
Total CPP baseline	3.26 × 10^4^ [1.49 × 10^4^–5.73 × 10^4^]	1.62 × 10^4^ [8.85E + 03–7.11 × 10^4^]	3.12 × 10^4^ [1.95 × 10^4−^6.12 × 10^4^]	*P* = 0.31
Total CPP 1 month	3.36 × 10^4^ [2.38 × 10^4^–5.70 × 10^4^]	1.59 × 10^4^ [1.18 × 10^4^–2.69 × 10^4^]	5.15 × 10^4^ [2.86 × 10^4^–1.47 × 10^5^]	*P* = 0.28
Total CPP 6 months	3.88 × 10^4^ [1.74 × 10^4^–6.44 × 10^4^]	1.84 × 10^4^ [1.08 × 10^4^–1.18 × 10^5^]	7.78 × 10^4^ [2.17 × 10^4^–1.65 × 10^5^]	*P* = 0.27
Total CPP 12 months	4.28 × 10^4^ [2.37 × 10^4^–6.28 × 10^4^]	2.02 × 10^4^ [1.15 × 10^4^–1.83 × 10^5^]	4 × 10^4^ [1.98 × 10^4^–1.42 × 10^5^]	*P* = 0.21
CPP-I baseline	9.18 × 10^3^ [4.01 × 10^3^–1.87 × 10^4^]	7.06 × 10^3^ [4.57 × 10^3^–2.065 × 10^4^]	1.81 × 10^4^ [5.1 × 10^3^–4.15 × 10^4^]	*P* = 0.52
CPP-I 1 month	1.64 × 10^4^ [7.08 × 10^3^–2.91 × 10^4^]	6.03 × 10^3^ [2.92 × 10^3^–4.53 × 10^4^]	4.39 × 10^4^ [1.63 × 10^4^–7.6 × 10^4^]	*P* = 0.11
CPP-I 6 months	1.25 × 10^4^ [6.8 × 10^3^–2.73 × 10^4^]	8.97 × 10^3^ [6.65 × 10^3^–2.82 × 10^4^]	2.78 × 10^4^ [1.28 × 10^4^–5.04 × 10^4^]	*P* = 0.53
CPP-I 12 months	1.41 × 10^4^ [× 10^3^–2.42 × 10^4^]	1.17 × 10^4^ [5.38 × 10^3^–7.04 × 10^4^]	1.47 × 10^4^ [1.26 × 10^3^–8.92 × 10^4^]	*P* = 0.14
CPP-II 0 months	1.81 × 10^4^ [5.78 × 10^3^–3.92 × 10^4^]	9.17 × 10^3^ [3.98 × 10^3^–5.07 × 10^4^]	9.37 × 10^3^ [7 × 10^3^–2.54 × 10^4^]	*P* = 0.45
CPP-II 1 month	2.02 × 10^4^ [7.5 × 10^3^–2.8 × 10^4^]	1.21 × 10^4^ [5.51 × 10^3^–2.25 × 10^4^]	1.23 × 10^4^ [7.67 × 10^3^–7.14 × 10^4^]	*P* = 0.81
CPP-II 6 months	1.63 × 10^4^ [8.27 × 10^3^–3.63 × 10^4^]	9.4 × 10^3^ [4.11 × 10^3^–8.93 × 10^4^]	1.49 × 10^4^ [3.7 × 10^3^–8.94 × 10^3^]	*P* = 0.74
CPP-II 12 months	2.57 × 10^4^ [1.02 × 10^4^–4.39 × 10^4^]	8.5 × 10^3^ [6.11 × 10^3^–1.12 × 10^5^]	1.36 × 10^4^ [9.94 × 10^3^–9.31 × 10^4^]	*P* = 0.36

Data presented as median [interquartile range]

Abbreviations: *CPP* calciprotein particles, *CPP-I* primary calciprotein particles, *CPP-II* secondary calciprotein particles

**Fig. 4:**
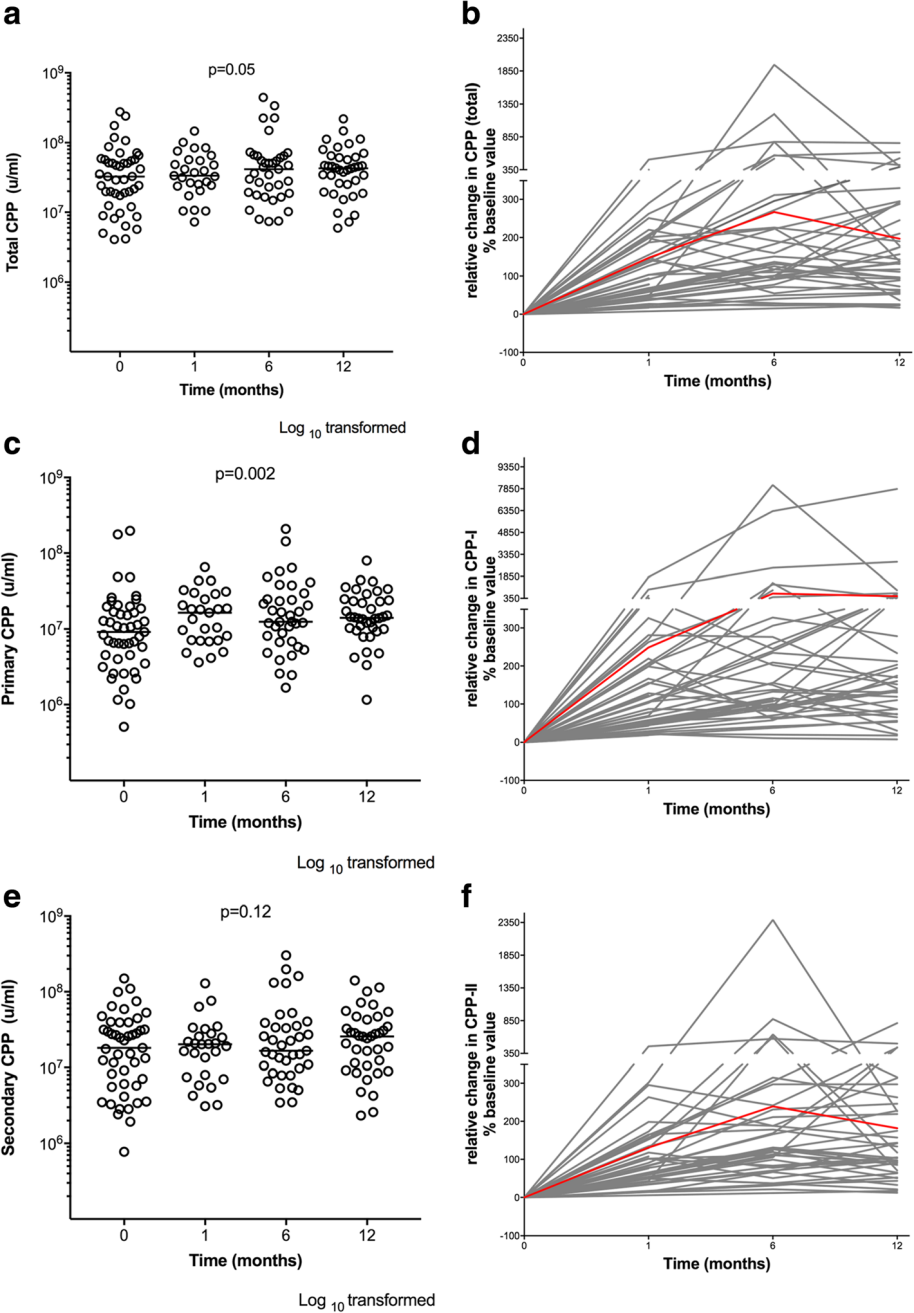
Changes in calciprotein particles over 12 months following cinacalcet withdrawal. Changes in absolute levels of (**a**) total CPP, (**c**) CPP-I and (**e**) CPP-II over a 12-month time period (median, lower and upper quartile, and outliers). P value denotes trend over 12-month period. Patient-level changes over study period expressed as relative percent change in (**b**) total CPP, (**d**) CPP-I and (**f**) CPP-II from baseline value with mean change highlighted in red over a 12-month period

CPP-I increased significantly over the study period (396% mean increase, *p* = 0.002). Total CPP showed a trend towards increasing levels, but this did not reach statistical significance (*p* = 0.05). Figure 4 depicts cohort and patient-level changes in serial absolute CPP levels over the study period and relative % changes from baseline values.

Utilizing a mixed linear effect model, allowing for random effects, changes in total and CPP-I counts were significantly associated with changes in PTH (*p* = 0.007 and 0.04 respectively). Total CPP, CPP-I and CPP-II counts were all longitudinally associated with changes in serum calcium (*p* = 0.002, 0.02 and 0.001 respectively), albumin (*p* = 0.001, 0.004 and < 0.001) and ferritin concentrations (*p* = 0.03, 0.01, and 0.009). Only changes in CPP-II were associated with the change in phosphate (*p* = 0.03) and ALP (*p* = 0.05). In contrast to those in the cinacalcet withdrawal cohort, there was no change in total CPP (*p* = 0.4), CPP-I (*p* = 0.2) or CPP-II (*p* = 0.1) in patients who received a kidney transplant or in those who re-started cinacalcet (total CPP (*p* = 0.15), CPP-I (*p* = 0.2), CPP-II (*p* = 0.2)) over the study period, although numbers were small.

**Fig. 5:**
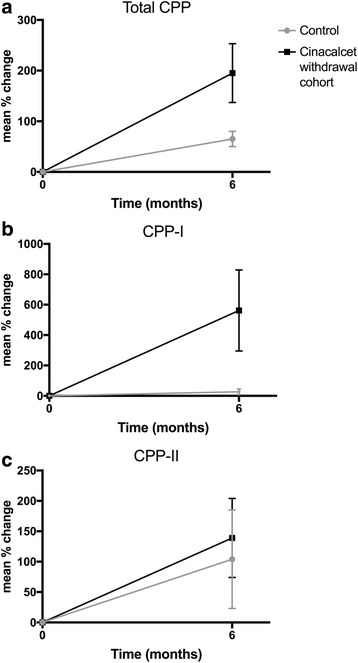
Mean percentage change and standard deviation in (**a**) total CPP, (**b**) CPP-I and (**c**) CPP-II over a six-month period in control dialysis cohort versus cinacalcet withdrawal patients. ^a^*P* < 0.001, ^b^*P* < 0.001, ^c^*P* = 0.24

Thirteen stable cinacalcet-naïve dialysis patients were used as a control cohort for CPP analysis, mean age 70.8±13years, 77% male, with 12 patients on hemodialysis and one on peritoneal dialysis. There were no significant differences in demographic characteristics between the control and the cinacalcet withdrawal cohorts, other than a higher proportion of patients on hemodialysis in the CPP analysis cohort (*p* = 0.02). There were no hospitalizations or changes in phosphate binder or active vitamin D prescription patterns in the control group. Over a six-month period, there was no significant increase in PTH (*p* = 0.1), serum calcium (*p* = 0.69), phosphate (*p* = 0.37), ALP (*p* = 0.86) or CPP-I (*p* = 0.08) and CPP-II (*p* = 0.26). In the control cohort of 13 stable cinacalcet naïve patients, CPP were mainly CPP-I (mean 79% CPP-I versus mean 21% CPP-II). The mean percentage increase in total CPP and CPP-I over a six-month period was significantly lower in the control group compared to the cinacalcet withdrawal cohort (*p* = 0.03 and *p* = 0.05 respectively). This effect was not seen for CPP-II (*p* = 0.07). Figure 5 shows the mean percentage changes in total CPP, CPP-I and CPP-II from baseline to 6 months values in cinacalcet withdrawal patients compared to control patients.

## Discussion

Whilst the EVOLVE study was inconclusive as to its effects on cardiovascular mortality overall, a major issue with EVOLVE was that it was underpowered to detect mortality in the age group recruited because of the low event rate in the control group [24]. For patients over the age of 65, where the parathyroidectomy and transplantation rates were much lower, the EVOLVE study did appear to derive a cardiovascular benefit from cinacalcet therapy. Nevertheless, whilst cinacalcet has been shown to be effective in controlling metabolic parameters, it cannot be recommended to improve survival without a better understanding of its link with cardiovascular mortality [25]. It would therefore be desirable to have a biomarker that could more directly illustrate the link between cardiovascular disease and SHPT. CPP appear to be such an emerging biomarker and therefore ideal for testing potential effects of cinacalcet removal.

Withdrawal in Australia of reimbursement for cinacalcet for the medical treatment of SHPT has enabled us to examine potentially important changes in this emerging biomarker associated with CKD-MBD. Cinacalcet cessation resulted in increased PTH, serum calcium, and CPP-I, which was associated with a reduction in serum albumin but no change in serum phosphate. Longitudinal changes in CPP were associated with an increase in PTH, calcium, ALP, ferritin and decrease in albumin.

There is mounting interest in the potential role of CPP in the pathogenesis of CKD-MBD. Recently our group published a novel validated method for CPP detection and quantitation [15] using a fluorescent probe-based flow cytometric assay, and this together with blood calcification propensity analysis [26] has shed new light on the potential involvement of these difficult-to-evaluate nanoparticles. The primary role of CPP-I is likely to act as a mineral chaperone, sequestering mineral that may otherwise seed mineralization at ectopic sites and facilitating transport and clearance from body fluids [27]. However, in states of chronic mineral stress, the transition and ripening of CPP-I to pro-inflammatory CPP-II may be enhanced, creating a vicious cycle of inflammation and calcification [13, 28].

Increased CPP has been associated with increased vascular calcification in animal models, and its presence in the serum of adenine-treated rats proceeds the developmental of vascular calcification [28]. CPP have also been identified in multiple other animal models including 5/6 nephrectomized rats, where there was an increase in CPP over a 10-week period of renal failure [14].

CPP have also been identified in humans [16, 20], with higher levels of CPP being predictive of all-cause mortality in CKD patients [17] as well as being associated with greater coronary artery calcification scores in dialysis patients [20] and increased aortic stiffness measured by pulse wave velocity in pre-dialysis CKD patients [18]. In our cinacalcet withdrawal group, we observed a 74% one-year survival rate compared to 80% in our control arm. While these survival rates are in keeping with the Australian and New Zealand Dialysis and Transplantation Registry (ANZDATA) reported one-year survival for the 65–74 year old age (the median age of our study population) of 87% [29], sample size and short follow-up preclude formal comparison of survival characteristics in these cohorts.

Whilst more research is needed to determine whether increases in CPP, and which subfraction, may be linked to outcome, we were able to show that although CPP increased over time in dialysis patients, the percentage change was significantly greater in cinacalcet withdrawal patients compared to stable cinacalcet naïve patients. We interpret the finding of enhanced CPP-I as indicative of increased mineral flux, potentially resulting from changes in bone turnover following cinacalcet cessation. Indeed, previous studies in rats suggest that bone is a likely source of circulating particles [30]. The strongly correlated changes of CPP-I with PTH and ALP over time, certainly support such a hypothesis. Presently, the potential clinical significance of this marked elevation in CPP-I is uncertain but since CPP-I transform into CPP-II under the influence of the uremic environment, higher levels of CPP-I may eventually engender increased CPP-II levels if persistent. Studies with longer follow-up are therefore needed to assess these effects. This may be important with respect to their putative pathogenicity as in vitro data clearly points to disparate effects of CPP-I and CPP-II on vascular smooth muscle cells [31].

The link between CPP and PTH has also been reported previously [14, 20]. Therapeutic interventions of cinacalcet or parathyroidectomy for the treatment of SHPT were shown to reduce fetuin-A mineral complexes (CPP) in dialysis patients. Our data is the first to report a rise in PTH is strongly associated with an increase in CPP. Most patients in the cinacalcet withdrawal group had a peak in CPP levels at 6 months with a plateau thereafter, which may well reflect attempts to control PTH levels with higher doses of activated vitamin D therapy. Some heterogeneity in the CPP trend was still observed and it is difficult to elicit whether this represents biological variation or the effects of interventions. The observation of a concomitant rise in ALP following cinacalcet withdrawal also contributes to the hypothesis of bone as a possible reservoir for these nanoparticles [20].

There was a reduction in calcium-based phosphate binder use in our study (*p* = 0.05) and a reduction in non-calcium-based phosphate binder use (*p* = 0.06) following cinacalcet withdrawal. One explanation for this may have been the concurrent reduction in calcitriol use following cinacalcet cessation (*p* = 0.03) as a result of rebound hypercalcemia. The reduction in calcitriol use may have contributed to a fall in phosphate absorption allowing for reduction in phosphate binder use, although this was an observed association and the rationale for this association is speculative.

Our study has several limitations and strengths. The novel method of identifying and quantifying CPP provides a greater understanding of their role in the mineralization paradigm. The methodology of CPP analysis in this study is novel and, although currently undertaken only by our team, this methodology is validated and published [15]. The prospective nature of the study and multiple time point measurements was useful in evaluating CPP trends over a 12-month period in the dialysis cohort and has not been performed to date. Unfortunately, samples for the cinacalcet-naïve control and study cohorts were collected and analysed at two different time points and comparison of absolute total CPP counts between these two populations is not possible due to nature of the analytical technique and inherent changes in laser optics over time. Presently, this limits the use of this assay to a research setting and makes it challenging to generate reference data for these nanoparticles in the dialysis population. However, comparison of relative changes remains valid and perhaps more informative given the order of magnitude of differences in absolute levels between individuals.

As our study is observational in nature, causality of the associations found, although likely, cannot be proven, but is useful in further hypothesis generation. This study was conducted in a single-center dialysis population, and therefore the sample size of the study population was small and likely contributed to the reduced magnitude of associations seen over a 12-month period. Unlike many observational studies that are cross-sectional in nature however, the strength of the current analysis is its longitudinal design allowing us to assess the evolution of changes in CPP and their inter-relationship with other biochemical parameters over time. Importantly despite the small sample size, we show significant temporal effects on CPP subsets and biochemically relevant co-correlations in serial comparisons.

## Conclusion

In conclusion, cinacalcet withdrawal in a dialysis population with SHPT was associated with an increase in PTH, serum calcium and CPP-I. Assessment of CPP using the novel fluorescent probe-based flow cytometric assay as described here, may provide further insight into the biochemical abnormalities associated with the CKD-MBD. Longer follow up of this population will allow us to determine if cinacalcet withdrawal is associated with increased rates of parathyroidectomies and higher cardiovascular mortality.

## Abbreviations

ALP: Alkaline phosphatase
CKD: Chronic kidney disease
CKD-MBD: Chronic kidney disease mineral bone disorder
CPP: Calciprotein particles
CRP: C-reactive protein
FGF23: Fibroblast growth factor 23
PTH: Parathyroid hormone
SHPT: Secondary hyperparathyroidism
